# GPR88 is a critical regulator of feeding and body composition in mice

**DOI:** 10.1038/s41598-017-10058-x

**Published:** 2017-08-30

**Authors:** Jackie Lau, Aitak Farzi, Ronaldo F. Enriquez, Yan-Chuan Shi, Herbert Herzog

**Affiliations:** 1 Neuroscience Division, Garvan Institute of Medical Research, St Vincent’s Hospital, Sydney, 2010 Australia; 2 Institute of Experimental and Clinical Pharmacology, Medical University of Graz, Graz, Austria; 3 Faculty of Medicine, UNSW Australia, Sydney, 2052 Australia

## Abstract

GPR88 is an orphan G-protein-coupled receptor with predominant expression in reward-related areas in the brain. While the lack of GPR88 has been demonstrated to induce behavioral deficits, the potential function of the receptor in the control of food intake and energy balance remains unexplored. In this work, the role of GPR88 in energy homeostasis was investigated in *Gpr88* ^−/−^ mice fed either standard chow or high fat diet (HFD). *Gpr88* ^−/−^ mice showed significantly reduced adiposity accompanied with suppressed spontaneous food intake, particularly pronounced under HFD treatment. While energy expenditure was likewise lower in *Gpr88* ^−/−^ mice, body weight gain remained unchanged. Furthermore, deregulation in glucose tolerance and insulin responsiveness in response to HFD was attenuated in *Gpr88* ^−/−^ mice. On the molecular level, distinct changes in the hypothalamic mRNA levels of *cocaine-and amphetamine-regulated transcript* (*Cartpt*), a neuropeptide involved in the control of feeding and reward, were observed in *Gpr88* ^−/−^ mice. In addition, GPR88 deficiency was associated with altered expressions of the anorectic *Pomc* and the orexigenic *Npy* in the arcuate nucleus, especially under HFD condition. Together, our results indicate that GPR88 signalling is not only important for reward processes, but also plays a role in the central regulatory circuits for energy homeostasis.

## Introduction

Obesity occurs when chronic increase in energy intake significantly exceeds energy expenditure^1^. Current pharmacological treatments for obesity, which primarily aim to suppress energy intake through reducing appetite or dietary fat absorption^2^, have demonstrated limited effectiveness and efficacy^3^ and can also lead to complex side effects^4^. The discovery of novel and effective pharmacotherapeutic targets is therefore a high priority.

G-protein-coupled receptors (GPCRs) represent the largest membrane receptor family in the mammalian genome, with over 800 genes identified in humans^5, 6^. Due to the fact that GPCRs are responding to various extracellular signals and thereby modulating diverse physiological processes^6^, more than 40% of clinically approved drugs target GPCRs to modulate their effects^7, 8^. Despite rapid research progress that led to the deorphanization of more than 300 GPCRs in the past two decades, over 150 GPCRs still remain orphans^9, 10^, among which a number were indicated as regulators of energy metabolism^11, 12^, hence offering therapeutic promises for obesity.

One of these orphan GPCR candidates is GPR88. This receptor is predominantly expressed in striatal projection neurons, with high evolutionary conservation between humans and rodents in both primary structure and expression pattern, suggesting critical functional conservation^13–16^. Associated with the reward network in the striatum, GPR88 has been implicated in numerous behaviors linked to neurological conditions in both rodents and humans, namely bipolar disorders, schizophrenia^17–19^, responses to psychostimulant drugs^20^ and antidepressants^21, 22^, as well as learning and social behavior^23, 24^. In addition to the abundant distribution throughout the striatum including the nucleus accumbens (Acb), caudate putamen (CPu) and olfactory tubercle, GPR88 expression is also enriched in inferior olive in the brainstem, cortex and amygdala^13–16, 25, 26^, with minimal to absent expression in peripheral tissues^13, 26, 27^. Interestingly, many of the GPR88-rich areas are involved in the reward processing system that is integrated and interrelated with the circuits controlling energy balance to direct downstream effects on appetite and food intake^28, 29^. The functional implication with the anatomical distribution of GPR88 expression suggests a potential role of the receptor in regulating energy homeostasis, an underexplored area of GPR88 research.

The emerging potential of GPR88 in energy homeostatic regulation is supported by several lines of evidence. First, mice lacking *Gpr88* globally showed over-represented striatal mRNAs that encoded proteins involved in cell response to nutrient levels and starvation, as revealed by microarray combined with gene ontology analysis^16^. In the same study, the most upregulated gene in the striatum of *Gpr88* knockout mice compared with controls was the cocaine- and amphetamine-regulated transcript (*Cartpt*)^16^, a key neuropeptide central to the control of appetite and energy balance^30^. Strikingly, microarray profiling in our *Cartpt* knockout mouse model^31^ revealed that *Gpr88* was amongst the most significantly upregulated GPCR mRNAs in the hypothalamus compared to wild-type mice, despite moderate hypothalamic expression of the receptor^13, 14, 25^. These correlative findings in the two genetic models indicate a potential function for GPR88 in the energy homeostatic circuit. Furthermore, plausible involvement of GPR88 in energy metabolism was denoted in an investigation of the sensitivity of individual abdominal white adipose depots to ghrelin exposure^32^, a gastric hormone that stimulates orexigenic hypothalamic neurons and plays a critical regulatory role in lipid storage and distribution^33, 34^. In contrast with earlier studies reporting the absence or minimal expression of *Gpr88* in peripheral tissues in rodents^26, 27^, *Gpr88* mRNA was identified to be more highly expressed in the ghrelin-unresponsive epididymal fat than the ghrelin-responsive retroperitoneal adipose tissue in rats^32^. The depot-specific *Gpr88* expression may attribute to differential energy utilization and lipid handling mechanisms^32^ involving this receptor. Concordantly, in a separate microarray study, *Gpr88* mRNA was upregulated in the hypothalamus following cold exposure in both leptin-deficient and wild-type mice^35^. The leptin-independent alteration in expression regardless of the nutritional status indicated possible association of GPR88 with the mobilization of fuel reserves in cold-activated thermogenesis^35^.

In this study, we aimed to elucidate the detailed function of GPR88 in the regulation of energy balance employing a germline *Gpr88* knockout mouse model. As previous characterizations of GPR88-deficient models focused mainly on behavioral parameters and psychiatric contexts^16, 20, 23, 25, 36, 37^, the present work offers the first insights into the physiological roles of this receptor in energy homeostatic control. The metabolic phenotypes as well as the underlying mechanisms were investigated. Effects of high fat diets on the manifestation of GPR88 deficiency were also addressed in comparison with standard chow feeds to examine the potential influence of *Gpr88* deletion on diet-induced obesity.

## Results

### Absence of GPR88 signalling has no effect on body weight gain

*Gpr88* ^−/−^ mice were born at the expected Mendelian ratios with equal gender ratio and litter size as controls (data not shown). In agreement with recent studies^16, 23^, no significant differences in body weight or appearance were observed at birth between these mice and their control littermates (data not shown). Both male and female mice were studied under chow conditions, while only male mice were subjected to HFD treatment. Based on the data from extensive metabolic characterization, male and female mice demonstrated consistency for the majority of the phenotypic traits, with greater prominence in phenotypes in males. Hence, data for male mice are the main focus, while key data for female mice are included as supplementary figures. In order to study the baseline metabolic characteristics of this *Gpr88* ^−/−^ model, and the effects of high fat dietary intervention, we first measured body weight (BW) gain and body composition under standard chow diet as well as HFD conditions.

**Figure 1:**
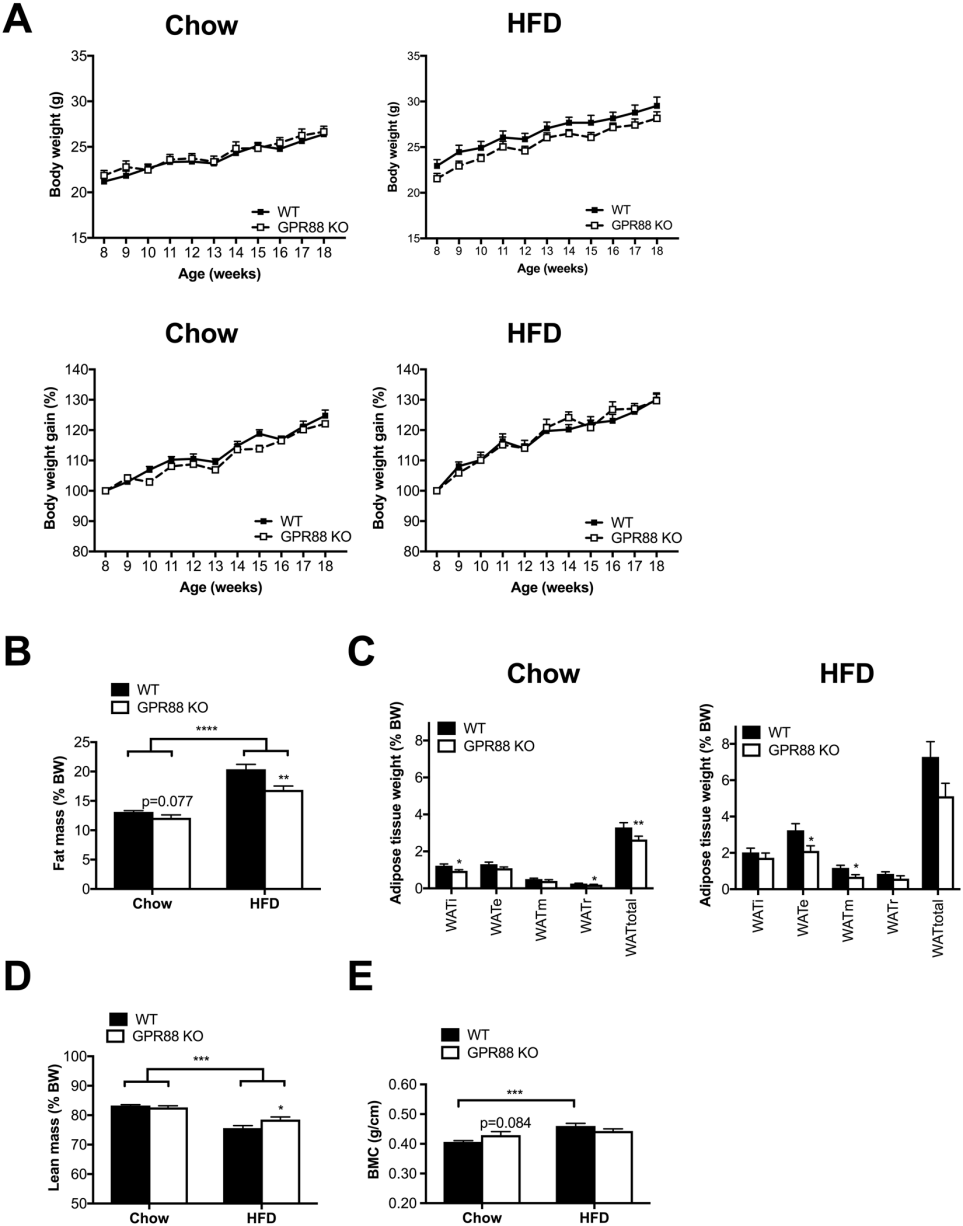
GPR88 deletion reduces adiposity under both diet conditions despite unaltered weight gain. (**A**) Absolute body weight (BW) (chow group) and BW change as a percentage of initial BW (chow and HFD groups) measured weekly in WT (n = 11) and *Gpr88* ^−/−^ (n = 10–12) mice. Whole body (**B**) fat mass and (**D**) lean mass as a percentage of body weight (%BW) as determined by body composition analysis using dual-energy X-ray absorptiometry (DXA). (**C**) Tissue mass (%BW) of dissected white adipose tissue (WAT) depots after sacrifice of mice. i, inguinal; e, epididymal; m, mesenteric; r, retroperitoneal; total, summed weight of i, e, m and r WAT depots. (**E**) Whole body bone mineral content (BMC) determined by DXA. Data are means ± SEM and averaged for all mice from each group examined. *p ≤ 0.05, **p ≤ 0.01, ***p ≤ 0.001, ****p ≤ 0.0001 for *Gpr88* ^−/−^ versus WT mice, or for comparisons between the same genotypes across dietary conditions.

*Gpr88* ^−/−^ mice on both chow and HFD gained similar body weight to WT control mice when expressed in absolute value as well as a percentage of initial body weight (Fig. 1A). As expected, HFD-fed *Gpr88* ^−/−^ and WT mice exhibited higher absolute body weight over time compared to mice on chow (Fig. 1A). However, there was no notable diet-related difference in percentage weight gain between mice from these two groups.

### Lack of GPR88 signalling results in increased lean mass and augmented loss in fat mass on HFD

To investigate the influence of GPR88 on body composition, DXA analysis was performed. For males from both diet groups, whole body fat mass expressed as a percentage of body weight was reduced in *Gpr88* ^−/−^ mice relative to WT controls at 16 weeks of age, significantly so under the HFD condition (Fig. 1B). This is consistently shown in female counterparts on chow (Figure S1). The lower fat mass in *Gpr88* ^−/−^ mice was also confirmed by decreased mass of dissected white adipose tissue depots for both genders (Figs 1C and S2). No observable difference was detected in the weights of major organs between genotypes despite dietary conditions or genders (Figure S3). In contrast to the reduction in adiposity, whole body lean mass (presented as a percentage of body weight) was significantly increased in *Gpr88* ^−/−^ mice compared to WT under HFD (Fig. 1D), but this was not seen in the chow cohort regardless of gender (Figs 1D and S4). As expected, other than greater baseline adipose masses (Fig. 1B,C), HFD-fed mice also exhibited lower baseline percentage lean mass with respect to chow-fed counterparts of the same genotypes (Fig. 1D). The coinciding of decreased fat mass and elevated lean mass in HFD-fed *Gpr88* ^−/−^ mice (Fig. 1B,D) may be one of the reasons for the resultant unchanged body weight gain (Fig. 1A).

### GPR88 signalling is involved in bone homeostasis under HFD conditions

In view of the variations in the body fat and lean contents, the influence of *Gpr88* ablation on bone mineral density and content was also examined via DXA scans. No significant genotype difference was observed for either total body BMC (Figs 1E and S5) or BMD (Figures S5 and S6) for mice from either diet or gender groups. Nevertheless, a tendency towards an increased BMC was shown for chow-fed *Gpr88* ^−/−^ mice compared with WT counterparts (Fig. 1E), which may possibly account for the unaltered weight gain (Fig. 1A) despite the lower fat mass in KO mice (Fig. 1B,C). In addition, femur length showed neither genotype- nor diet-related difference (Figure S7). However, consistent with previous reports^38, 39^ and as expected, WT mice displayed significantly greater BMC (Fig. 1E) and BMD (Figure S6) when fed a HFD compared to standard chow. Interestingly, for *Gpr88* ^−/−^ mice, the HFD-induced increase in mineralization was only apparent for BMD (Figure S6) while absent for BMC (Fig. 1E), indicating that mice lacking GPR88 may exhibit altered responsiveness to the effects of high-fat feeding on specific bone parameters.

### GPR88 deficiency reduces food intake and energy expenditure independent of diet

**Figure 2:**
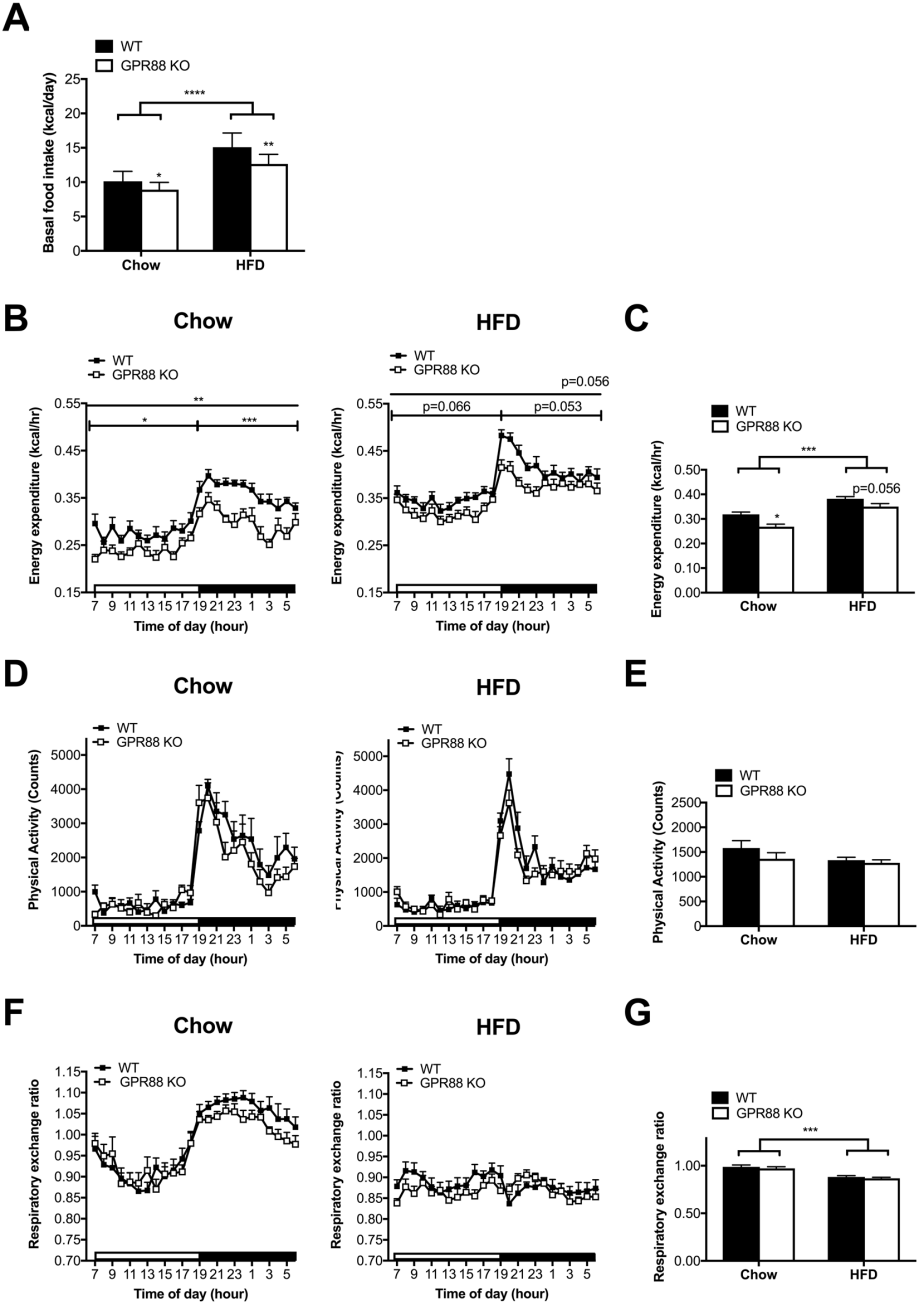
GPR88 deficiency diminishes food intake and energy expenditure under both diet conditions despite unchanged physical activity and predominant fuel source. (**A**) Daily spontaneous/basal food intake during fed state expressed as kilocalorie (kcal) in WT (n = 11) and *Gpr88* ^−/−^ (n = 10–12) mice under standard chow and HFD feeds. Indirect calorimetric assessments for the 24-hr time course of energy expenditure (**B**,**C**), physical activity (**D**,**E**) and respiratory exchange ratio (**F**,**G**). Corresponding average values for each parameter over the total 24-hr period were shown in adjacent bar graphs. Energy expenditure was adjusted for lean mass and compared between groups by analysis of covariance. The adjusted means of energy expenditure were presented at the common lean mass of 20.3 g (chow) and 20.926 g (HFD) respectively. Open and filled horizontal bars indicate the light and dark photoperiods, respectively. Data are means ± SEM and averaged for all mice from each group examined. *p ≤ 0.05, **p ≤ 0.01, ***p ≤ 0.001, ****p ≤ 0.0001 for *Gpr88* ^−/−^ versus WT mice, for comparisons between the same genotypes across dietary conditions, or for comparisons indicated by horizontal bar.

In light of the altered body composition, particularly reduced body fat mass, in *Gpr88* ^−/−^ mice, we also investigated the effects of GPR88 deficiency on energy intake and energy expenditure^1, 29, 40^. Interestingly, 24-hr spontaneous food intake was significantly decreased in *Gpr88* ^−/−^ compared to WT mice regardless of dietary conditions or genders (Figs 2A and S8), probably contributing to the marked decline in fat mass seen in *Gpr88* ^−/−^ mice across diets (Figs 1B,C and S1–2). Consistent with the substantially lower whole body fat mass (Fig. 1B), the reduction in food intake in KO mice relative to WTs was more significant when put on HFD compared to chow (Fig. 2A).

We also examined the other side of the energy balance equation and investigated energy expenditure using open-circuit indirect calorimetry. A substantial decrease in energy expenditure was shown in *Gpr88* ^−/−^ males relative to WT controls on both diets (Fig. 2B,C), significantly so under a standard chow condition, throughout the entire photoperiod and particularly prominent during the dark phase. Such significant decrease was absent in female *Gpr88* ^−/−^ counterparts (Figure S9). This reduced energy expenditure in male *Gpr88* ^−/−^ mice may represent a compensatory response to the decreased food intake (Fig. 2A), resulting in an unaltered overall weight gain between genotypes (Fig. 1A). It is noteworthy that the lesser drop in energy expenditure in HFD-fed KO mice (Fig. 2B,C) correlated with the greater loss of body fat mass in the same mice (Fig. 1B) compared with chow-fed counterparts. However, no significant changes were seen in physical activity for both genders (Figs 2D,E and S10), indicating that the decreased energy expenditure in *Gpr88* KO males was not due to any alteration in physical activity on either diet. Similarly, RER, an indicator of metabolic fuel selection, was comparable between *Gpr88* ^−/−^ and WT mice under both diet conditions for both genders (Figs 2D,E and S11), suggesting GPR88 may not be involved in oxidative fuel preference. As expected, HFD-fed males of both genotypes showed a significantly lower RER compared with chow-fed counterparts (Fig. 2G), signifying an enhanced fuel source preference for fat over carbohydrate, likely due to the higher adiposity accrual available under a state of energy surplus.

### GPR88 signalling may be involved in feeding under fasting conditions

**Figure 3:**
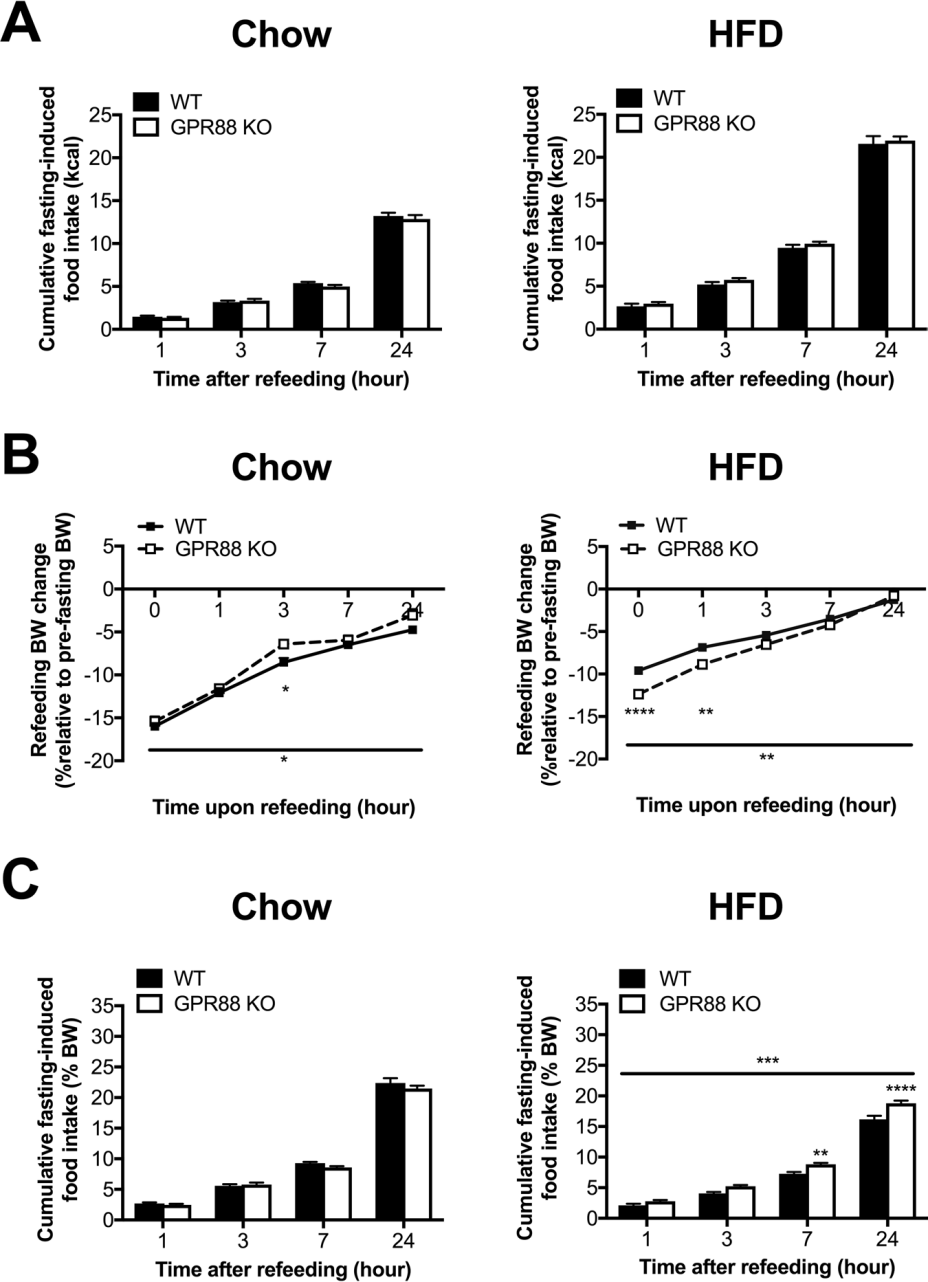
Absence of GPR88 promotes fasting-induced feeding under HFD. Cumulative 24-hr fasting-induced food intake during fasted state expressed as kcal (**A**) and as a percentage of body weight (**C**) in WT (n = 11) and *Gpr88* ^−/−^ (n = 10–12) mice under standard chow and HFD feeds. (**B**) The corresponding BW change in proportion to pre-fasting BW measured at the time points of 24-hr fasting-induced food intake experiments. Data are means ± SEM and averaged for all mice from each group examined. *p ≤ 0.05, **p ≤ 0.01, ***p ≤ 0.001, ****p ≤ 0.0001 for *Gpr88* ^−/−^ versus WT mice, or for comparisons indicated by horizontal bar.

Additional to evaluating feeding control during the fed state, the impact of GPR88 ablation on food intake was explored at a state of energy deficit induced by fasting. When expressed as absolute caloric energy values, there was no observable difference in fasting-induced food intake between genotypes on either chow or HFD treatment (Figs 3A and S12). In response to 24-hr fast, while the relative weight loss with respect to pre-fasting BW was unaltered between genotypes for the chow cohort (Figs 3B and S13), HFD-fed KO mice experienced substantially greater proportion of weight loss compared to WT (Fig. 3B). Furthermore, *Gpr88* ^−/−^ mice exhibited accelerated BW recovery throughout refeeding with respect to WT controls under both diets, although this was only seen in males (Figs 3B and S13). This pattern of improved weight recovery was more dramatic in HFD cohort, with similar proportion of BW between genotypes at 24 hr after refeeding (Fig. 3B). Interestingly, when expressed as a percentage of body weight, fasting-induced food intake was comparable between *Gpr88* ^−/−^ and WT mice under the chow condition regardless of genders (Figs 3C and S14). However, the genotype difference became statistically significant when challenged with HFD, with significantly increased fasting-induced feeding in *Gpr88* ^−/−^ mice compared to WT controls (Fig. 3C), particularly at 7- and 24-hr time points following refeeding.

### GPR88 knockout mice show differential expression of key hypothalamic peptides in the control of energy balance

**Figure 4:**
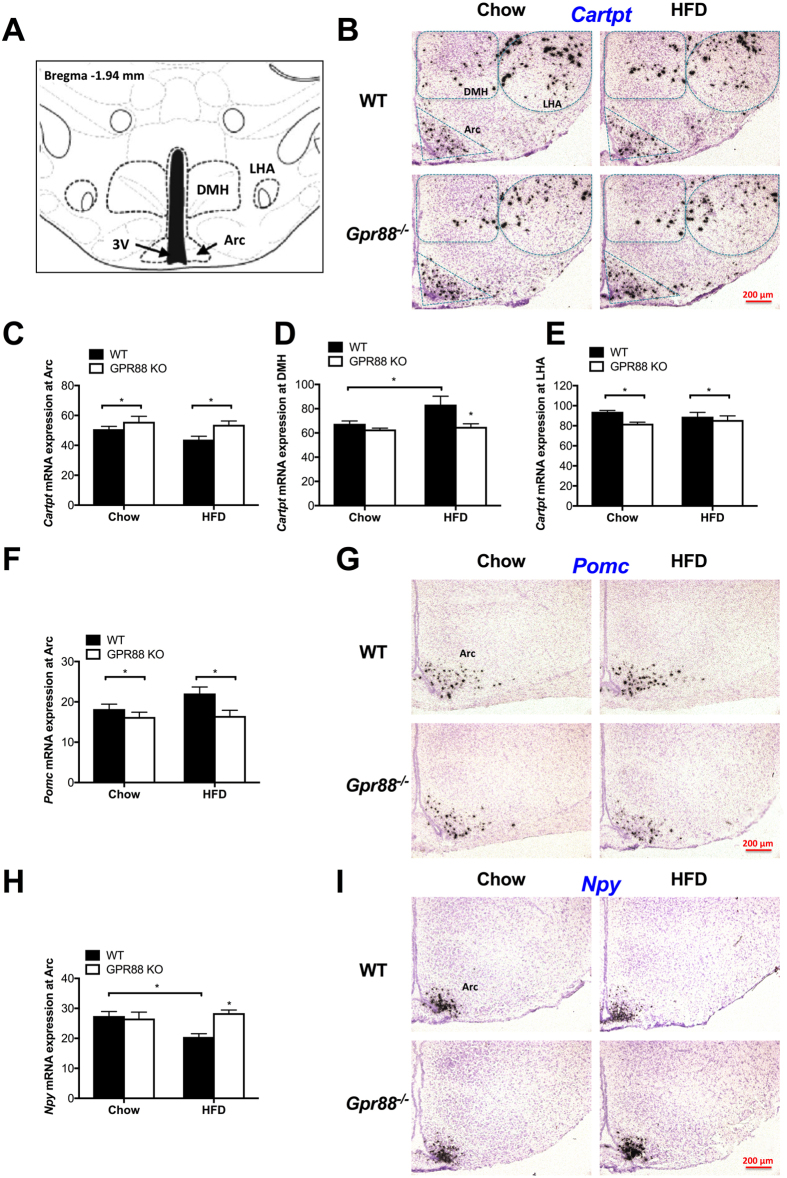
GPR88 ablation alters the hypothalamic expression of key neuropeptides in feeding control and energy homeostatic. (**A**) Schematics indicating the specific level of selected coronal brain sections with respect to the bregma. Representative bright-field photomicrographs depicting mRNA expression of *Cartpt* in the DMH, LHA and Arc (**B**), and of *Pomc* (**G**) and *NPY* (**I**) in the Arc. Scale bar = 200 µm. *In situ* hybridization for *Cartpt* mRNA expression at the Arc (**C**), DMH (**D**), and LHA (**E**), as well as for *Pomc* (**G**) and *Npy* (**I**) mRNA expression at the Arc in WT (n ≥ 7) and *Gpr88* ^−/−^ mice (n ≥ 5) on standard chow and HFD treatments. Hybridization signals are quantified to obtain mean labelling intensity of neurons expressed as percentage coverage of neuronal surface by silver grains (RODs) within the defined areas of interest. Mean RODs for *Cartpt* in the (**C**) Arc – chow: WT (50.85), KO (55.75) (difference = 4.90); HFD: WT (43.81), KO (53.76) (difference = 9.95). Mean RODs for *Cartpt* in the (**D**) DMH – chow: WT (67.52), KO (62.88) (difference = 4.64); HFD: WT (83.37), KO (65.06) (difference = 18.31). Mean RODs for *Cartpt* in the (**E**) LHA – chow: WT (93.93), KO (82.03) (difference = 11.90); HFD: WT (89.04), KO (85.76) (difference = 3.28). Mean RODs for *Pomc* in the (**F**) Arc – chow: WT (18.22), KO (16.27) (difference = 1.96); HFD: WT (22.09), KO (16.51) (difference = 5.58). Mean RODs for *Npy* in the (**H**) Arc – chow: WT (27.45), KO (26.58) (difference = 0.87); HFD: WT (20.47), KO (28.38) (difference = 7.91). 3 V, third ventricle; Arc, arcuate nucleus; DMH, dorsomedial hypothalamic nucleus; LHA, lateral hypothalamic area. Data are means ± SEM and averaged for all mice from each group examined. *p ≤ 0.05 for *Gpr88* ^−/−^ versus WT mice, or for comparisons indicated by horizontal bar.

To elucidate the central mechanisms underlying the *GPR88*-deficient phenotypes manifested under the two dietary conditions, hypothalamic expression of several neuropeptides pivotal in appetite control and energy metabolism was profiled. *In situ* hybridization was performed to determine the transcript expression of *Cartpt, Pomc* and *Npy* in the Arc (Fig. 4A), the two sets of first-order neurons that respond to circulating adiposity signals and traditionally classified as anorexigenic and orexigenic, respectively^41–43^. Messenger RNA levels of *Cartpt* were also examined in the DMH and LHA of the hypothalamus (Fig. 4A,B), CART-rich regions also known to be involved in feeding behaviors and weight regulation^44–48^. Significant increase in *Cartpt* mRNA level was observed in the Arc of *Gpr88* ^−/−^ mice relative to WT controls (Fig. 4C), more prominently under HFD condition. In contrast, *Cartpt* expression in the DMH was significantly lower in *Gpr88* ^−/−^ than WT mice on HFD (Fig. 4D), while the decrease was less notable in chow-fed KOs. In the LHA, where second-order effector neurons receive projections from the Arc^43, 44^, *Cartpt* expression was pronouncedly downregulated in *Gpr88* ^−/−^ mice compared to WT (Fig. 4E), with greater effect under chow condition.

Contrary to the upregulated Arc *Cartpt* (Fig. 4C), mRNA levels of the colocalized *Pomc* in the Arc^49, 50^ (Fig. 4F,G) showed a distinct decline in *Gpr88* ^−/−^ relative to WT mice, more substantially when put on HFD (Fig. 4F). Interestingly, the opposite was shown for the expression of the adjacent Arc *Npy* (Fig. 4H,I), which was significantly upregulated in HFD-fed *Gpr88* ^−/−^ mice only (Fig. 4H), although no difference was detected between genotypes for the chow fed cohort. The expression changes in *Gpr88* ^−/−^ mice on HFD were generally more prominent compared with chow-fed counterparts for all three neuropeptides at the hypothalamic areas examined (Fig. 4C,D,F,H), with the exception of LHA *Cartpt* (Fig. 4E).

With regard to diet effects, baseline expressions of both *Npy* (Fig. 4H) and *Cartpt* (Fig. 4C) in the Arc were both notably downregulated in WT controls when fed HFD compared to standard chow, significantly so for *NPY*. In contrast, notable upregulation was shown for Arc *Pomc* (Fig. 4F) and significantly for DMH *Cartpt* (Fig. 4D) in WT mice on HFD relative to chow diet. Remarkably, the prominent diet-induced effects were absent in GPR88-deficient mice regardless of the genes or areas of interest (Fig. 4), indicating potential resistance to certain neurochemical consequences following chronic high-fat feeding in the absence of GPR88.

### GPR88 deficiency alleviates deregulation in glucose tolerance and insulin responsiveness associated with HFD treatment

**Figure 5:**
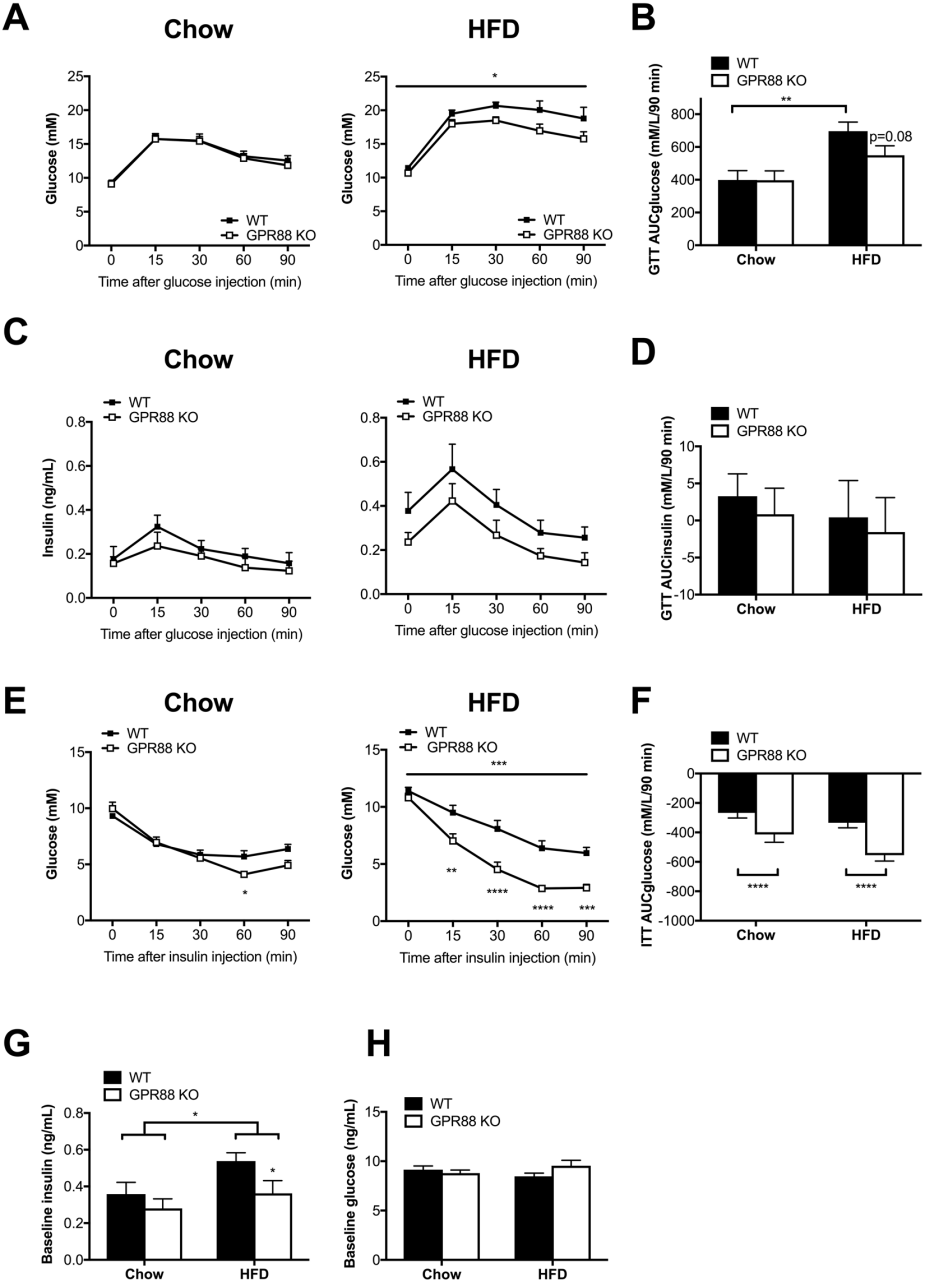
GPR88 deficiency offers relief from HFD-induced impairment in glucose tolerance and insulin responsiveness. (**A**) Blood glucose levels during a 90-min intraperitoneal glucose tolerance test (1.0 g/kg body weight) performed in 6-hr fasted WT (n = 11) and *Gpr88* ^−/−^ (n = 10–12) mice under standard chow and HFD conditions. (**B**) Area under the glucose concentration curve (AUCglucose) between 0 and 90 min after glucose injection. (**C**) Corresponding serum insulin levels during the 90-min IPGTT procedure. (**D**) Area under the insulin concentration curve (AUCinsulin) between 0 and 90 min after glucose injection. (**E**) Absolute blood glucose levels during a 90-min intraperitoneal insulin tolerance test (0.5 IU/kg body weight) performed in 5-hr fasted WT and *Gpr88* ^−/−^ mice. (**F**) AUCglucose between 0 and 90 min after insulin injection. Baseline insulin (**G**) and glucose (**H**) levels during fed state. Data are means ± SEM and averaged for all mice from each group examined. *p ≤ 0.05, **p ≤ 0.01, ***p ≤ 0.001, ****p ≤ 0.0001 for *Gpr88* ^−/−^ versus WT mice, for comparisons between the same genotype across dietary conditions, or for comparisons indicated by horizontal bar.

In light of the well-established link between fat metabolism and glucose homeostasis^51^, and based on the decreased fat content in *Gpr88* knockout mice, various parameters of glucose metabolism were investigated. Following i.p. glucose challenge, blood glucose levels throughout the 90 min duration were indistinguishable between WT and *Gpr88* ^−/−^ mice on chow (Fig. 5A). In contrast, when fed a HFD, *Gpr88* ^−/−^ mice demonstrated significantly lower blood glucose levels compared with WT during the course of IPGTT (Fig. 5A), despite comparable basal levels upon 6-hr fasting and similarly shaped blood glucose response curves. Although both genotypes showed markedly greater blood glucose levels when put on HFD, *Gpr88* ^−/−^ mice exhibited significantly improved glucose clearance relative to WT (Fig. 5A). Concordantly, the relatively enhanced glucose response indicated for *Gpr88* ^−/−^ mice on HFD was reflected in the strong trend of reduction in the area under the glucose curve with respect to WT (Fig. 5B). Such genotype difference in AUCglucose was absent in the chow cohorts (Fig. 5B). Interestingly, despite differential blood glucose responses in HFD group, no significant difference was shown in the corresponding serum insulin levels during IPGTT between *Gpr88* ^−/−^ and WT mice under both diet conditions (Fig. 5C). The lack of substantial difference was also shown in the area under the insulin curve (AUCinsulin) (Fig. 5D). However, while similarly shaped patterns of insulin response were observed, KO mice on HFD appeared to exhibit a trend of dampened glucose-stimulated insulin secretion compared to WT counterparts (Fig. 5C). This suggests that lower insulin release may be sufficient to efficiently eliminate glucose in GPR88-deficient mice.

Insulin responsiveness was examined using the IPITT test. While chow-fed WT and *Gpr88* ^−/−^ mice displayed comparable blood glucose levels in response to IPITT (Fig. 5E) during 0–30 min, KO mice demonstrated a generally steeper decline, where the blood glucose levels became significantly decreased at the lowest point at 60 min compared to WT (Fig. 5E). Interestingly, when put on HFD, *Gpr88* ^−/−^ mice showed a significant decrease in blood glucose throughout the 90 min period compared with WT, with similar initial fasted blood glucose levels at 0 min (Fig. 5E), as reflected by the corresponding AUCglucose (Fig. 5F). Consistent with insulin levels from IPGTT at a 6-hr fasted state, plasma insulin levels in *Gpr88* ^−/−^ mice during the control fed state were unchanged on chow diet, and significantly lower on HFD, when compared to WT (Fig. 5G). Unlike the higher baseline insulin seen in mice of both genotypes on HFD relative to chow (Fig. 5G), no difference was detected for baseline glucose levels between genotypes or diet groups (Fig. 5H). The improved glucose tolerance in *Gpr88* KO mice on HFD was thus attributed to the greater insulin responsiveness, which may be secondary to the higher lean mass^52^ observed compared to WT (Fig. 1D).

## Discussion

While considerable efforts have been made to explore the role of GPR88 in reward and behavioral aspects, our work provides the first evidence that GPR88 also influences the regulation of energy homeostasis at various levels. This is clearly demonstrated in mice lacking GPR88, which showed a significant decrease in adiposity associated with marked reduction in daily spontaneous food intake under chow and high fat diet conditions, without any change in body weight gain. This was also accompanied by a distinct decline in energy expenditure in *Gpr88* ^−/−^ mice compared with controls, with unchanged physical activity and RER. At an energy-deficit state following fasting, refeeding-food intake was substantially increased in *Gpr88* ^−/−^ mice fed a HFD while unaltered for chow-fed counterparts. Moreover, when challenged with HFD, *Gpr88* ^−/−^ mice showed a significant increase in lean mass. This, together with decreased fat mass, could contribute at least partially to the improved insulin responsiveness and thus glucose tolerance seen in *Gpr88* ^−/−^ mice on HFD, which showed unaffected insulin secretion. We also demonstrated that lack of GPR88 has less effect on bone metabolism. Mechanistically, *Cartpt* expression in *Gpr88* ^−/−^ mice on HFD was increased in the Arc and reduced in the DMH respectively, in line with the anorectic phenotype. However, since lower *Pomc* and higher *Npy* mRNA levels in the Arc were also displayed in the same mice, CART may play a more critical role in determining the feeding behavior in GPR88-deficient mice.

The effects of *Gpr88* deletion on feeding behavior and body composition consistently suggested the orexigenic potential of this receptor. Although the impact of *Gpr88* ablation on appetite regulation was unexplored in previous studies^16, 20, 23, 25, 36, 37^, mice lacking *Gpr88* were reported to show reduced novelty-suppressed feeding^37^. The altered feeding behaviour^37^ possibly attributed to the impaired cue-based learning shown in a separate study in a *Gpr88* ^−/−^ mouse model identical to that used in the present work^16^, hence indirectly endorsing the decreased basal food intake in the fed state observed herein. Interestingly, in this study, *Gpr88* ^−/−^ mice exhibited decreased spontaneous food intake but increased fasting-induced food intake under HFD, indicating differential roles of GPR88 in the regulation of food consumption under varying states of energy status. Since WT mice typically experience less weight loss following energy deprivation when fed a HFD relative to chow^53^, the advantage in weight reservation conferred by a fat-rich diet may be abolished in the absence of GPR88.

In the present study, we demonstrated a decrease in energy expenditure in *Gpr88* ^−/−^ mice, which predicts an increased body weight gain^40^. However, this effect was potentially cancelled out by the lower food consumption, leading to an overall unaltered weight gain in *Gpr88* ^−/−^ mice. In spite of an unchanged weight gain in *Gpr88* ^−/−^ mice, the body composition was altered, such that body fat mass was increased and lean mass was reduced when challenged with HFD. The augmented drop in adiposity in *Gpr88* ^−/−^ mice on HFD than chow suggested that GPR88 may function as part of the biological defense of elevated body fat during obesity development^29^. Interestingly, despite the lower energy expenditure, no change was observed in the RER and physical activity, although other groups have reported involvement of the receptor in locomotor activity and responses^16, 37, 54^. The discrepancy may be explained partly by the distinct age- and gender-factors involved, as well as the different methods of measurements, as the majority of literature on GPR88 adopted behavioral tests. In addition, alterations in GPR88 expression in various brain regions have been documented in WT mice under various stress conditions and environments^55^, affecting diverse cognitive and emotional processing^36^. Accordingly, manifestation of GPR88-deficiency on different settings may engage distinctive behavioral and motor responses.

The wide expression of GPR88 in the brain^13, 14, 54^, together with the evident alterations in aspects of energy homeostasis on a *Gpr88*-negative background, suggested modulations in the central circuits for energy balance. Intriguingly, despite predominant expression in the striatum^13, 14, 54^, latest single cell RNA sequencing data revealed minute levels of *Gpr88* expression in *Npy*- and *Cartpt/Pomc*-containing neurons in the mouse hypothalamus^56^. In line with this, results from the present work also distinctively support the novel association between GPR88 and specific hypothalamic neuropeptides implicated in appetite and weight regulation. First, differential *Cartpt* mRNA expression was displayed in *Gpr88* ^−/−^ mice at various feeding-related regions, namely a general upregulation in the Arc and downregulation in the DMH and LHA under both diet conditions, in line with the reduced feeding observed. Despite controversies surrounding CART function in feeding control^30^, increase in the classically anorexigenic Arc *Cartpt* levels may contribute to the suppressed basal food intake in *Gpr88* KO, both parameters with greater genotype difference when fed a HFD. Additionally, among other hypothalamic regions, DMH and LHA have been considered orexigenic or hunger centers, as lesions in each area individually led to hypophagia and weight loss^57–59^. Since heightened *Cartpt* transcript levels in the DMH and LHA were linked to elevation in feeding and body weight^60^, the anorectic effects seen in *Gpr88* KO may also attribute to the declined *Cartpt* expression in these two areas, both with indicated involvement in the orexigenic activity of CART^46, 60, 61^. The varying hypothalamic changes in *Cartpt* expression in *Gpr88* KO, namely higher in the Arc and lower in the DMH and LHA, hence collectively endorse the inhibited spontaneous food intake, which indirectly led to reduced fat mass and increased lean mass, particularly when challenged with HFD treatment. The results conjointly support a novel role of GPR88 as an orexigenic signal. Contrastingly, in spite of the substantially higher expression of the orexigenic *Npy* and lower levels of the anorexigenic *Pomc* in the Arc, KO mice demonstrated a hypophagic phenotype. Whilst the suppressed *Pomc* levels may represent a counter-regulatory response to the elevated Arc *Cartpt*, CART effects on the overall food intake of *Gpr88* ^−/−^ mice were suggested to be stronger than impacts from the altered expression of either *Npy* or *Pomc*. Nevertheless, other possible mechanisms together with the regulations in hypothalamic neuropeptides may have effectively counteracted the anorexigenic outcome of *Gpr88* deficiency, resulting in comparable weight gain between genotypes on both diets. GPR88 is thus indicated as a novel component of the complex interplay between the orexigenic or anorexigenic pathways that fine-tune feeding behavior. Further studies characterizing the extensive neurochemical profile of *Gpr88* KO mice will provide valuable insights into the inferred function for the receptor.

In conclusion, this work is the first direct evidence for a physiological role of GPR88 in the regulation of energy balance. The data suggest that under a chow-diet condition, GPR88 may be important in the regulation of food intake, adiposity, energy expenditure and bone mineralization. However, active involvement of the receptor was not indicated in other metabolic aspects, such as physical activity or metabolic fuel selection. Under conditions of diet-induced obesity, *Gpr88* ablation improved body lean content, weight recovery and feeding following fasting, insulin responsiveness and glucose tolerance. The metabolic changes were mechanistically associated with *Cartpt* expression that was increased in the Arc and diminished in the DMH respectively. GPR88 in mice is thus proposed to promote fat deposition, enhance appetite as well as energy expenditure. While the manifestation of GPR88 effects may be augmented by dietary manipulation, such as in a state of chronic energy surplus induced by high-fat feeding, ablation of *Gpr88* alone was insufficient to influence overall body weight. In other words, stimulation of GPR88 signalling is conjectured to trigger hyperphagia and increase susceptibility to obesity, whereas such obesogenic drive may be counteracted by feedback regulatory mechanisms efficiently to boost metabolism and prevent excessive adipose deposition. This is supported by the dichotomous alterations of hypothalamic neuropeptides with anorexigenic and orexigenic properties in GPR88-deficient mice, implicating an interactive element to GPR88 in the system of energy homeostatic efficiency. Future research elucidating the pharmacology and neurochemical dynamics of GPR88 will shed light on the therapeutic potential of this orphan receptor in obesity treatment. Findings from this study suggest that targeting GPR88 could have beneficial effects in modulating fat accretion, feeding behavior, and glucose homeostasis in obesity-related comorbidities.

## Materials and Methods

### Animals and grouping

All research and animal care procedures were approved by the Garvan Institute/St. Vincent’s Hospital Animal Ethics Committee and were in agreement with the Australian Code of Practice for the Care and Use of Animals for Scientific Purposes. The *Gpr88* knockout mice (*Gpr88* ^−/−^) were a generous gift from Prof. Richard Palmiter (University of Washington, USA), with targeting strategies described in detail previously^16^. All mice were housed under conditions of controlled temperature of 22 °C and illumination (12:12 hr light-dark cycle, lights on at 07:00 hr). Mice were provided with *ad libitum* access to water and either standard chow diet (8% calories from fat, 21% calories from protein, 71% calories from carbohydrate, 2.6 kilocalorie (kcal)/g; Gordon’s Specialty Stock Feeds, Yanderra, NSW, Australia) or high fat diet (HFD, 23% calories from fat, 19.4% calories from protein, 48.2% calories from carbohydrate, 4.7% calories from crude fibre, 4.7% calories from acid detergent fibre, 4.78 kcal/g; Gordon’s Specialty Feeds, Glen Forrest, WA, Australia) according to grouping. Both male and female mice were used for all experiments under chow conditions, whereas only male mice were subjected to HFD treatment.

All mice were initially fed a standard laboratory chow diet. At 8 weeks (wks) of age, subsets of mice from both the *Gpr88* knockout (KO; *Gpr88* ^−/−^)^16^ and wild-type (WT) groups were subjected to HFD feeding for 10 wk until sacrifice at 18 wk of age. The remaining subsets of mice continued on the standard chow diet. The same experimental procedures were performed on both chow- and HFD-fed groups in an age-matched manner.

### Determination of body weight and food intake

All *Gpr88* ^−/−^ and WT mice fed on standard chow diet or HFD were subject to the same experimental procedures as follows. Body weight was measured weekly throughout the duration of studies. Mice were assessed for spontaneous/basal food intake in the fed state as well as for fasting-induced food intake in response to 24-hr fasting at 12 wks of age. In brief, mice were transferred from group housing on soft bedding to individual cages with paper towel bedding for 3 nights of acclimatization. Basal daily food intake was determined as the average of duplicate readings obtained over two consecutive 24-hr periods. Twenty four-hr fasting-induced food intake was subsequently measured at 1, 3, 7, and 24 hr after refeeding with the respective types of diet, while the corresponding body weight was recorded in parallel.

### Indirect calorimetry of energy expenditure and fuel source preference

All mice from both chow- and HFD-fed groups were evaluated for metabolic parameters and physical activity at 16 wk of age. For energy metabolism, metabolic rate was measured by indirect calorimetry using an 8-chamber open-circuit calorimeter (Oxymax series; Columbus Instruments, Columbus, OH, USA). Mice were housed individually in specially built Plexiglas cages (20.1 × 10.1 × 12.7 cm). Temperature was maintained at 22 °C with airflow of 0.6 L/min. Mice were singly housed for 3 days in home cages prior to acclimatization in Plexiglas cages for 24 hr before commencement of recording. Monitoring was subsequently performed for 24 hr in the metabolic chambers. Mice were provided with *ad libitum* access to water and food of respective diet types. Body weight was measured and food given in excess was weighed before and after the recording period for the quantification of daily food intake after subtraction of spillage. Oxygen consumption (*V*O_2_) and carbon dioxide production (*V*CO_2_) were measured every 27 min. The respiratory exchange ratio (RER) was calculated as the quotient of *V*CO_2_/*V*O_2_, with 100% carbohydrate oxidation represented by the value of 1.0 and 100% fat oxidation the value of 0.7. Energy expenditure (kcal heat produced) was determined as calorific value (CV) x *V*O_2_, where CV is 3.815 + 1.232 x RER. Data obtained for the 24-hr monitoring period were averaged at 1-hr intervals for energy expenditure and RER.

### Body composition and bone densitometry analysis

All mice from both chow- and HFD-fed groups were subjected to an initial body composition analysis using the dual-energy X-ray absorptiometry (DXA, Lunar PIXImus2 mouse densitometer; GE Healthcare, Waukesha, WI, USA) system at 10 wk of age. A second DXA scan was performed 6 wk after initial DXA measurements at 16 wk of age, immediately following completion of the indirect calorimetry assessment of energy metabolism and physical activity. Animals were anesthetized with isoflurane for the scanning procedure to determine whole body bone mineral density (BMD), bone mineral content (BMC), fat and lean mass, where lean mass corresponds to non-fat and non-bone tissue content. The head of the animal was excluded while the tail included for the analysis.

### Glucose metabolism studies

All mice from both chow- and HFD-fed cohorts were examined by intraperitoneal glucose tolerance test (IPGTT) and insulin tolerance test (IPITT). IPGTT and IPITT were conducted at 14 and 17 wk of age respectively. For IPGTT, mice were subjected to 6-hr fasting where food was removed from cage hoppers at 09:00 hr and injection of a dose of a 10% D-glucose solution (1.0 g/kg body weight; Astra Zeneca, North Ryde, NSW, Australia) was performed 6 hr later into the peritoneal cavity. For IPITT, mice were subjected to 5-hr fasting where food was removed from cage hoppers at 10:00 hr and injection of a dose of insulin (0.5 IU/kg body weight) (Novo Nordisk Pharmaceuticals, Baulkham Hills, NSW, Australia) was performed 5 hr later into the peritoneal cavity. For both tests, tail vein blood was collected at 0, 15, 30, 60, 90 min following glucose or insulin injection and glucose concentrations were measured using a glucometer (Accu-Check II; Roche, Castle Hill, NSW, Australia). For IPGTT, insulin levels were subsequently quantified using a sensitive rat insulin radioimmunoassay (RIA) kit (Millipore, Billerica, MA, USA). Glucose and insulin tolerance curves constructed for the glucose levels obtained during IPGTT and IPITT respectively are presented in terms of absolute values. Area under the glucose (AUCglucose) or insulin (AUCinsulin) concentration curves between 0 and 90 min after glucose injection during IPGTT were plotted after subtraction of the initial concentrations prior to injection.

### Tissue collection

Following completion of studies, all mice from both chow- and HFD-fed groups were sacrificed at 18 wk of age. Animals were culled between 13:00 to 17:00 hr through cervical dislocation followed by decapitation. Brains were promptly collected and frozen on dry ice, then stored at −80 °C until subsequent analysis as described below. The white adipose tissue (WAT) depots from the inguinal, epididymal or periovarian (gonadal), retroperitoneal and mesenteric regions were removed and weighed. Tissue weights are normalized and expressed as a percentage of body weight.

### *In situ* hybridization analysis of major hypothalamic neuropeptides

*In situ* hybridization was conducted on brain sections of WT (n ≥ 7) and *Gpr88* ^−/−^ (n ≥ 5) mice from chow- and HFD-fed groups to determine mRNA expression of *Cartpt*, proopiomelanocortin (*Pomc*) and neuropeptide Y (*Npy*) at the hypothalamus following procedures as previously described^31, 62^. Briefly, matching coronal brain sections (25 µm) collected from the WT and *Gpr88* ^−/−^mice were prepared from specific brain levels with respect to the bregma to represent the hypothalamic areas involved in the regulation of energy homeostasis. Brain sections from mice of both genotypes and diet groups were processed in parallel for quantitative comparison. Detection of the mRNA expression was performed for *Cartpt*, *Npy*, and *Pomc* in the arcuate nucleus (Arc), as well as *Cartpt* in the dorsomedial hypothalamic nucleus (DMH) and lateral hypothalamic area (LHA). The cryosections were hybridized with [α-^35^S]-thio-dATP (PerkinElmer, Boston, MA, USA) radiolabelled DNA oligonucleotides complementary to mouse *Cartpt* (5′-TCCTTCTCGTGGGACGCATCATCCACGGCAGAGTA GATGTCCAGG-3′), *Npy* (5′-GAGGGTCAGTCCACACAGCCCCATTCGCTTG TTACCTAGCAT-3′), and *Pomc* (5′-TGGCTGCTCTCCAGGCACCAGC TCCACACATCTATGGA-GG-3′). Hybridization signals on sections were visualized by exposure to BioMax MR film (Kodak, Rochester, NY, USA) for 7–10 days and digitalized images from the scanned autoradiograms were acquired. For structural details, the brain sections were photoemulsion-dipped and superficially counterstained with haematoxylin, with which regions of interest were visualized and captured into digital images acquired by bright-field microscopy. Quantification of the mRNA expression levels of respective genes was performed by measuring the relative optical densities (RODs) within the brain areas of interest outlined with consistent defined dimensions across corresponding sections on the photomicrographs using the National Institutes of Health ImageJ 1.61 software (written by Wayne Rasband; available from anonymous FTP at zippy.nimh.nih.gov). Background labelling was considered uniform with signal levels below 5% of specific signal levels and was subtracted from the resultant signal density. Data are evaluated and presented as percentage of ROD averaged from at least three sections per mRNA assessed per animal.

### Statistical analysis

All data are presented as means ± SEM. Differences amongst mouse groups of various genotypes and dietary treatments were assessed by ANOVA or repeated-measures ANOVA combined with Bonferroni post-hoc analysis where appropriate. Energy expenditure, RER and physical activity over the continuous 24-hr period were averaged for the whole 24-hr period, as well as the 12-hr light and dark phases individually. Comparison of energy expenditure (kcal/hr) between groups was performed by the analysis of covariance (ANCOVA) with lean mass as the covariate. The adjusted means of energy expenditure at a common lean mass for the comparison were generated by ANCOVA as presented. Statistical analyses were performed with GraphPad Prism 6 for Mac OS X (GraphPad Software, Inc. CA, USA) and SPSS for Mac OS X version 16.0.1 (SPSS Inc., Chicago, IL, USA). Statistical significance was defined as *p*-value ≤ 0.05.

### Data availability

The datasets generated and analysed during the current study are available from the corresponding author on reasonable request.

## Electronic supplementary material

Supplementary figures
