# Supplementary material for: GPR88 is a critical regulator of feeding and body composition in mice
Source: Sci Rep. 2017 Aug 30;7:9912. doi: 10.1038/s41598-017-10058-x (PMC5577241; doi:10.1038/s41598-017-10058-x)
Supplement: Supplementary file 1 — Supplementary figures [file 41598_2017_10058_MOESM1_ESM.pdf]

GPR88 is a critical regulator of feeding and body composition in mice

Jackie Lau[1], Aitak Farzi[1,2], Ronaldo F. Enriquez[1], Yan-Chuan Shi[1,3,*] & Herbert Herzog[1,3*,]

[1]Neuroscience Division, Garvan Institute of Medical Research, St Vincent's Hospital, Sydney, 2010, Australia.

[2]Institute of Experimental and Clinical Pharmacology, Medical University of Graz, Graz, Austria.

[3]Faculty of Medicine, UNSW Australia, Sydney, 2052, Australia.

[*]Equal senior authors

Address correspondence to: Prof. Herbert Herzog

Dr. Yan-Chuan Shi

Neuroscience Division

Garvan Institute of Medical Research

384 Victoria Street, Darlinghurst NSW 2010

Sydney, Australia

Tel: 61 2 9295 8296

Fax: 61 2 9295 8281

Email: h.herzog@garvan.org.au

y.shi@garvan.org.au

Supplementary figures

S1

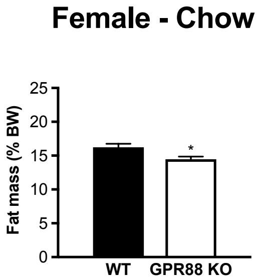

S2

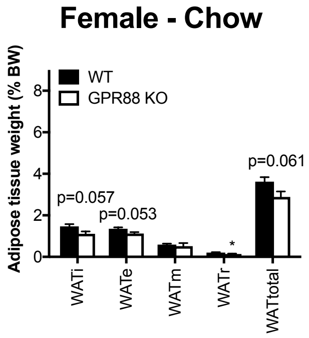

S3

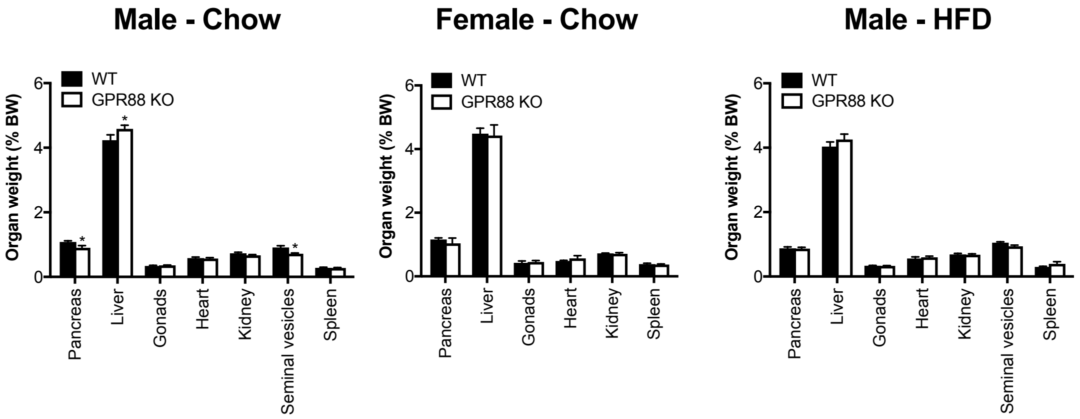

S4

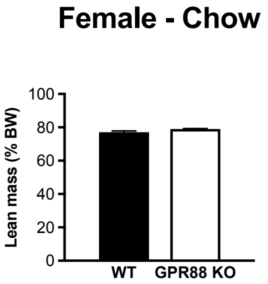

S5

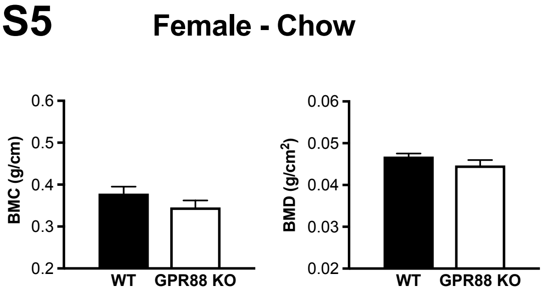

S6

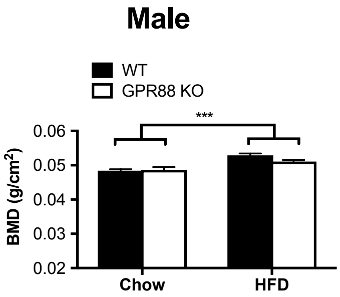

S7

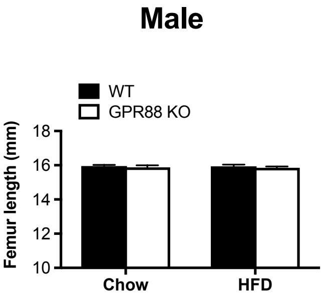

Figures S1 – S7: GPR88-deficient mice show reduced adiposity, unaltered weights of major organs and unchanged bone phenotypes. Whole body (**S1**) fat mass and (**S4**) lean mass as a percentage of body weight (%BW) as determined by body composition analysis using DXA in WT (n = 6) and *Gpr88*$^{-/-}$ (n = 5-6) female mice on standard chow. (**S2**) Tissue mass (%BW) of dissected white adipose tissue (WAT) depots after sacrifice of mice. i, inguinal; e, epididymal; m, mesenteric; r, retroperitoneal; total, summed weight of i, e, m and r WAT depots. (**S3**) Mass (%BW) of key organs after sacrifice of female mice on chow, as well as male WT (n = 11) and *Gpr88*$^{-/-}$ (n = 10-12) mice on chow and HFD. (**S5**) Whole body bone mineral content (BMC) and density (BMD) determined by DXA in chow-fed female mice. (**S6**) Whole body BMD in male mice under both diet conditions. (**S7**) Femur length determined with a Vernier caliper measuring the distance from the highest point of the first trochanter to the deepest point of the internal condyle in male mice. Data are means ± SEM and averaged for all mice from each group examined. *$p \leq 0.05$, ***$p \leq 0.001$ for *Gpr88*$^{-/-}$ versus WT mice, or for comparisons between the same genotypes across dietary conditions.

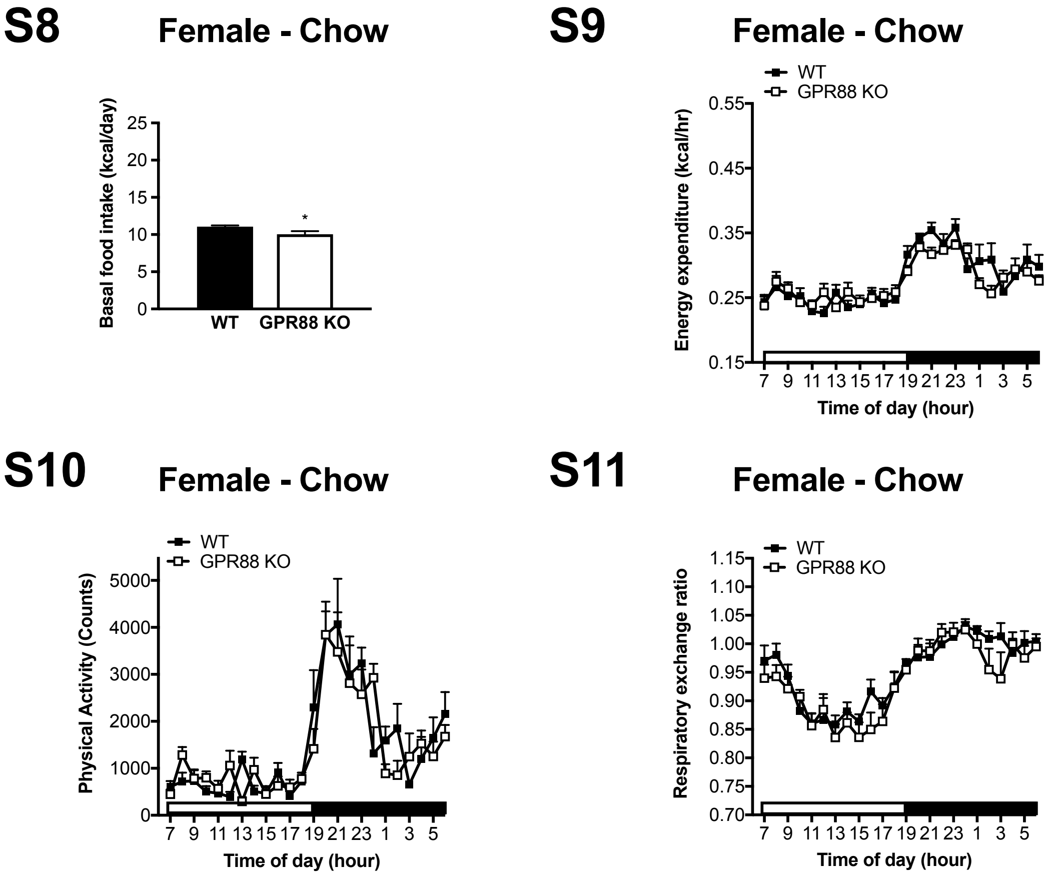

Figures S8 – S11: GPR88 deficiency diminishes food intake despite unchanged energy expenditure and physical activity in female mice. (**S8**) Daily spontaneous/basal food intake during fed state expressed as kilocalorie (kcal) in WT (n = 5) and *Gpr88*$^{-/-}$ (n = 5-6) female mice under standard chow feeds. Indirect calorimetric assessments for the 24-hr time course of energy expenditure (**S9**), physical activity (**S10**) and respiratory exchange ratio (**S11**). Energy expenditure was adjusted for lean mass and compared between groups by analysis of covariance. The adjusted means of energy expenditure were presented at the common lean mass of 16.027 g. Open and filled horizontal bars indicate the light and dark photoperiods, respectively. Data are means ± SEM and averaged for all mice from each group examined. *$p \leq 0.05$ for *Gpr88*$^{-/-}$ versus WT mice.

S12

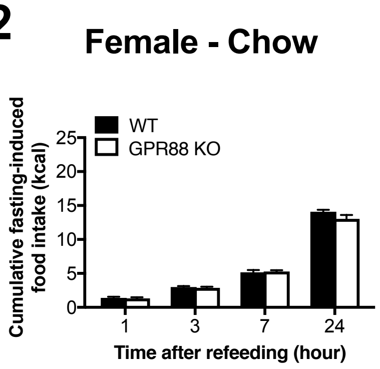

S13

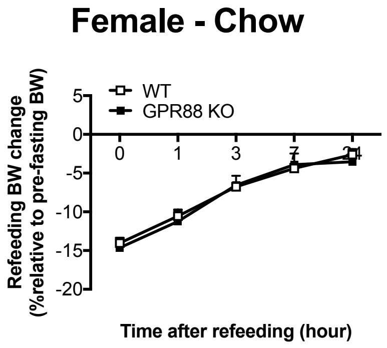

S14

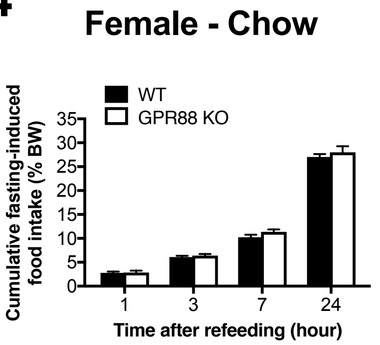

Figures S12 – S14: Absence of GPR88 has no effects on fasting-induced feeding in female mice. Cumulative 24-hr fasting-induced food intake during fasted state expressed as kcal (**S12**) and as a percentage of body weight (**S14**) in WT (n = 5) and *Gpr88*$^{-/-}$ (n = 5-6) female mice under standard chow feeds. (**S13**) The corresponding BW change in proportion to pre-fasting BW measured at the time points of 24-hr fasting-induced food intake experiments. Data are means ± SEM and averaged for all mice from each group examined.
